# Uniting to end the TB epidemic: advances in disease control from prevention to better diagnosis and treatment

**DOI:** 10.1186/s12916-016-0599-1

**Published:** 2016-03-23

**Authors:** Ibrahim Abubakar, Marc Lipman, Timothy D. McHugh, Helen Fletcher

**Affiliations:** Centre for Infectious Disease Epidemiology and MRC Clinical Trials Unit, University College London, London, UK; UCL-TB, University College London, London, UK; Division of Medicine, University College London, London, UK; Centre for Clinical Microbiology, University College London, London, UK; Department of Immunology and Infection, London School of Hygiene and Tropical Medicine, London, UK; TB Centre, London School of Hygiene and Tropical Medicine, London, UK

**Keywords:** Elimination, Multi-drug resistant tuberculosis, Tuberculosis

## Abstract

Tuberculosis is a major global cause of morbidity and mortality. Despite recent advances in containing the epidemic, several challenges continue to slow progress towards elimination including the continuing impact of drug resistant disease, and the lack of appropriate tools. Curtailing the transmission of tuberculosis remains a challenge especially in high burden countries. New developments in measuring correlates of protection are urgently needed to support the evaluation of vaccines. Similarly, despite progress in molecular diagnostics, better tools are required to identify resistance to antibiotics in multi and extensively drug resistant tuberculosis. Whole Genome Sequencing may lead to the next generation of assays to rapidly detect resistance and evaluate transmission. Advances on shortening treatment are hampered by the lack of a biomarker of cure which obviates the current long wait for relapses in trials. New research is urgently needed to support development of new vaccines and better diagnostics tools and shorter treatment for drug sensitive and resistant tuberculosis.

## Editorial

Tuberculosis (TB) is a leading global cause of morbidity and mortality due to an infectious agent [1]. The World Health Organization (WHO) estimates that levels of TB have been declining [1]. Unfortunately, multi drug resistant tuberculosis (MDR-TB) has emerged as a major problem globally with nearly half a million cases of MDR-TB diagnosed annually [1].

The WHO developed The End TB Strategy [2] as a response to the global TB epidemic. In keeping with the sustainable development goal (SDG) ambition to rid the world of major infectious disease killers including TB, HIV and malaria, the End TB Strategy has a vision of a world free of tuberculosis – zero deaths, disease and suffering due to TB by 2035. The strategy has 3 pillars; integrated patient centered care, bold policies and support systems and intensified research and innovation.

To mark this year’s World TB Day which has the fitting theme “Unite to end TB” the journals *BMC Medicine* and *BMC Infectious Diseases*, in collaboration with the TB centres at the London School of Hygiene and Tropical Medicine and University College London, have developed a collection of articles summarizing key advances and gaps in knowledge on TB. Published primary studies and reviews in this series include transmission studies, the diagnosis of TB and associated drug resistance, treatment and reports investigating immune correlates and the development of vaccines.

## Transmission

Transmission of *Mycobacterium tuberculosis* (Mtb) especially in high burden settings is a major problem [3]. While our understanding of several aspects of TB transmission has improved over the last two decades due to innovative technology such as molecular fingerprinting, geographical information systems, improved tests for TB infection, cough studies, mathematical modelling and other analytical tools, much remains poorly elucidated. The occurrence of TB is driven by HIV co-infection, increasing levels of co-morbidities such as diabetes, suboptimal housing and living standards and social deprivation. However the extent to which each of these factors determine the global trend is less clear. In low incidence, high income countries, the key drivers of TB include homelessness, drug misuse, history of imprisonment and disease occurring in migrants from high TB incidence countries [4, 5].

Developments such as whole genome sequencing (WGS) with its potentially greater resolution in distinguishing linked cases, are beginning to provide a greater insight into important questions such as the genetic relatedness of strains allowing the more efficient investigation of outbreaks. Witney and colleagues provide examples of local and published incidents and outbreaks where WGS has been used to improve the discriminatory ability of existing technology and to provide further information on missed transmission events [6]. By contrast, Hatherell and colleagues systematically reviewed studies using WGS to investigate transmission, outlining several areas of uncertainty. The limitations outlined were affected by the level of diversity, the depth of sequencing, available epidemiological data, prevalence of TB and therefore consequent level of new infection versus re-infection as well as mixed infection versus the rate of microevolution [7]. Both reviews identified the need for further research including the development of better bioinformatics tools.

In a study examining contact patterns between adults, youths and children in different settings in Zambia and South Africa, McCreesh and colleagues, found marked variation in the type of building where contact happens and the extent of mixing of age groups [8]. While these data are limited to specific sites in two high burden settings, they suggest the need to consider evaluating such information prior to any intervention.

## Biomarkers and immune correlates for tuberculosis vaccine development

There is an urgent need for an effective vaccine to protect against adult, pulmonary TB disease if we are to reduce transmission of TB and accelerate the current decline in global TB incidence. Immune correlates could help to accelerate TB vaccine development through 1) identification of promising TB vaccine candidates in pre-clinical or early clinical development 2) for targeted recruitment of individuals at risk of development of TB disease into efficacy trials 3) accurate identification of trial participants who are infected with TB 4) for early identification of participants who develop TB disease 5) as a substitute for clinical efficacy and 6) to ultimately further our understanding of the vaccine inducible immune mechanisms important for protection from TB disease.

Progress is being made in the development of tools for screening pre-clinical vaccine candidates based on functional ability to restrict the growth of mycobacteria rather than any single immune component. Examples of this include the mycobacterial growth inhibition assay (MGIA) which can also be used in early phase human clinical trials for identification of promising vaccine candidates. The ability to detect a vaccine effect against TB infection or TB disease is limited by our ability to accurately diagnose these states. Haas and colleagues describe how transcriptomics, proteomics and metabolomics are aiding the accurate and rapid diagnosis of TB infection and active TB disease [9]. In addition to aiding clinical decisions such markers can be used in TB vaccine trials to increase the accuracy of a TB infection or disease end point. Infants vaccinated with BCG have highly diverse immune responses, which reflect underlying differences in monocyte quantity and phenotype. These differences may impact on TB vaccine efficacy and should be taken into account during efficacy testing of candidate TB vaccines.

## Diagnosis, drug resistance and treatment

In many settings, smear microscopy, chest x-ray and bacterial culture remain the mainstay of TB diagnosis. While recent advances in molecular diagnostics are changing the landscape for the diagnosis of TB, the accuracy of such tests in many subpopulations such as children is still poor. The response to emergence of drug resistance in an individual, or indeed at a programmatic level, requires robust information about the drug resistance profile of the *Mtb* strain involved. Obtaining this information by conventional microbiology is too slow to be of use, but the advent of molecular tools has facilitated a rapid, though imperfect picture of resistance. The importance of an indication of drug sensitivity is highlighted by the paper by Martin and colleagues, which demonstrates that the selective use of molecular tools can lead to a significant under identification of drug resistance and the concomitant failure to treat adequately [10]. The authors call for investment in services to allow the microbiological diagnosis to be completed. Of course tools such as the Xpert MTB/RIF, have a lot to offer in this respect; but this provides evidence of rifampicin resistance and presumptive evidence of multi-drug resistance.

The increased accessibility of WGS has begun to change the diagnostic landscape, at least in high income settings. Thus, Witney and colleagues describe how WGS methods are being adapted for diagnostic use, including rapid identification of single nucleotide polymorphisms (SNPs) associated with known drug resistance [6]. This approach is already in use to inform the management of patients with complex drug resistance patterns in the absence of timely phenotypic tests. However, the authors rightly point to the areas that require development not least the need for a robust and user friendly bioinformatics pipeline. A key piece of information that is required to underpin this is the ability to correlate drug resistance with a SNP. It is not always possible to identify the necessary phenotype for sequence analysis and so Phelan and colleagues present a modelling approach to map potential resistance genotypes [11]. Using protein structure prediction they are able to predict polymorphisms associated with resistance. They propose that this approach can be used to inform the development of novel diagnostics with improved predictive value for phenotypic drug resistance.

Thus, we see that appropriate diagnosis of drug resistance, like TB diagnosis generally, is an area in development. The power of molecular tools needs to be applied carefully, but the emerging use of WGS and the associated analysis offers a real opportunity to provide rapid and robust drug sensitivity data in a timely fashion.

Treatment for drug sensitive TB remains stubbornly at six months despite efforts to improve upon this. Microbiological indicators of treatment response that can discriminate between different regimens and also predict long term clinical outcome at an individual patient level would be of great value. The REMoxTB trial, which compared two 4 month regimens containing moxifloxacin to standard 6 month treatment and where cultures were obtained weekly for 8 weeks and monthly thereafter, provides an opportunity to assess the performance of a variety of different measures of sputum culture conversion. Phillips and colleagues show that whilst there is an advantage in using continuous endpoints (which capture time on treatment) better than measuring the proportion of negative cultures at a given time, none were discriminatory enough to be used as either a trial surrogate endpoint or as an indicator of individual patient outcome [12].

Phillips and colleagues showed that patient factors were often most important in predicting outcome. The study by Ranzani and colleagues, based in Sao Paulo State, Brazil, highlights this. Here, successful treatment outcome was directly related to an individual’s social circumstances – with less than half of the homeless population studied having a favourable outcome (43 % compared to 83 % in the comparator group) [13]. This was mainly due to large numbers being lost to follow up or dying. Although the number of homeless subjects was relatively small at 3 %, they were a population with high rates of HIV co-infection, and hence had considerable health needs beyond TB. As is frequently the case, this study emphasises the importance of an engaged and co-ordinated societal response if we are to control TB. In this collection of articles, Phillips and colleagues describe an innovative approach, the “STEP trial design”, which may accelerate regimen development by ensuring a move towards simpler and shorter later phase evaluation of drug regimen [14].

## Conclusion

Ending the global TB epidemic by 2035 will require research to develop the tools to accelerate progress beyond that possible with current diagnostics, treatment regimen, vaccines and public health measures. The studies published in this series demonstrate some of the progress that has been made to date. However, they also illustrate the gaps requiring continued and reinvigorated funding if we are to achieve the SDG goal of ridding the world of TB.

## Abbreviations

MDR-TB: Multi drug resistant tuberculosis
MGIA: Mycobacterial growth inhibition assay
Mtb: *Mycobacterium tuberculosis*
SDG: Sustainable development goal
SNPs: Single nucleotide polymorphisms
TB: Tuberculosis
WGS: Whole genome sequencing
WHO: World Health Organization
